# A new and potentially prebiotic α-cytidine derivative†
†Electronic supplementary information (ESI) available. CCDC 1528414. For ESI and crystallographic data in CIF or other electronic format see DOI: 10.1039/c7cc00693d



**DOI:** 10.1039/c7cc00693d

**Published:** 2017-02-23

**Authors:** Maria Tsanakopoulou, Jianfeng Xu, Andrew D. Bond, John D. Sutherland

**Affiliations:** a MRC Laboratory of Molecular Biology , Francis Crick Avenue , Cambridge Biomedical Campus , CB2 0QH , UK . Email: johns@mrc-lmb.cam.ac.uk; b Department of Chemistry , University of Cambridge , Lensfield Road , CB2 1EW , UK

## Abstract

A new α-cytidine derivative was synthesised from the prebiotic reaction of ribose aminooxazoline and dicyanoacetylene. The tetracyclic structure of the product was confirmed by X-ray diffraction and then an alternative 6-step synthetic pathway to the product was found which was suitable for large-scale synthesis.

## Abstract 2

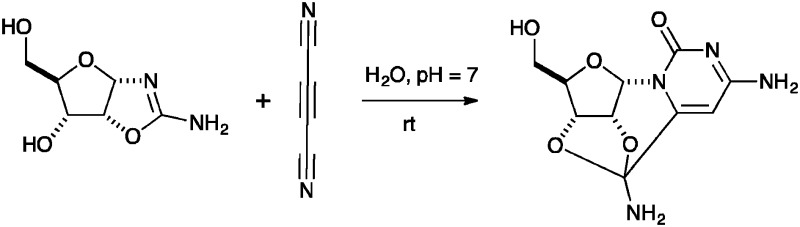
A new α-cytidine derivative was synthesised from the prebiotic reaction of ribose aminooxazoline and dicyanoacetylene.

In seminal prebiotic chemistry studies which prompted our follow-on work, Sanchez and Orgel reported that reaction of ribose aminooxazoline **1** with cyanoacetylene **2** gives a *ribo*-configured anhydronucleoside **3** which subsequently hydrolyses to give α-cytidine **4** (Scheme 1).^1^ Their efforts to extend this sequence of reactions into a plausible synthesis of the canonical pyrimidine nucleotides were thwarted, however, by their inability to photoanomerize α-cytidine **4** in anything more than 4% yield, and by difficulties in synthesizing ribose from which **1** was generated by condensation with cyanamide. We subsequently uncovered two prebiotically plausible syntheses of the canonical pyrimidine nucleotides involving pentose aminooxazoline intermediates made by the addition of 2-aminooxazole to glyceraldehyde.^2,3^ Our first synthesis proceeded *via* arabinose aminooxazoline and involved stereoinversion of C-2′,^2^ the second proceeded *via* ribose aminooxazoline **1** and involved high-yielding photoanomerisation of α-2-thiocytidine.^3^

**Scheme 1:**
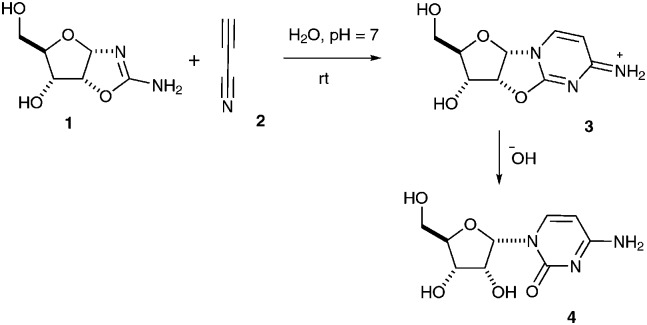
Reaction of ribose aminooxazoline **1** with cyanoacetylene **2**.

Although both ribose and arabinose aminooxazolines are formed in comparable yields in our synthetic routes, the *ribo*-configured material **1** has the advantage over the *arabino*-isomer in that it crystallises out of the mixture of reaction products and residual starting materials. Not only does this crystallisation allow for a spontaneous chemical purification of ribose aminooxazoline **1**, it also amplifies any enantiomeric excess initially present in solution because this highly crystalline compound is a conglomerate.^4^ This behaviour could have contributed to the formation of enantiopure RNA at the origin of life. Given the foregoing and the need for a prebiotically plausible synthesis of purine nucleotides – previous efforts being low yielding or of questionable prebiotic plausibility^5^ – we wondered if these latter canonical nucleoside derivatives might also derive from ribose aminooxazoline **1**. Since ribose aminooxazoline **1** functions as a nucleophile, we began to consider other electrophiles with which it might react to give adenosine and guanosine precursors. As it happens, this line of thinking has not (yet) resulted in a synthesis of the purine nucleotides, but it led serendipitously to the results described herein and so we relate it here. In particular, we were drawn to electrophiles which were synthetically related to cyanoacetylene **2** which we showed could be produced as its copper(i) complex, CuC_3_N, by copper(ii) promoted oxidative coupling of hydrogen cyanide and acetylene.^6^ Therefore, it did not seem unreasonable to consider the potential product of a further copper(ii) promoted oxidative coupling between CuC_3_N and hydrogen cyanide, which would be dicyanoacetylene **5**. Although this latter compound could not form a stable σ-complex with Cu(i), it could potentially form a π-complex. In addition, **5** has been detected by IR spectroscopy in Titan's atmosphere.^7^ Although we have not demonstrated a synthesis of dicyanoacetylene **5** by oxidative coupling, we now report that the reaction of ribose aminooxazoline **1** with **5** furnishes a new α-cytidine derivative, which was analysed by NMR spectroscopy and X-ray diffraction. This compound was then made by another, more conventional synthetic route in 6 steps starting from α-cytidine **4**.

The potentially prebiotic reaction of ribose aminooxazoline **1** with dicyanoacetylene **5** was performed at room temperature in aqueous solutions at pH = 6.5–7.0 and was monitored by ^1^H NMR spectroscopy. The only product we could isolate from the reaction mixture was a white crystalline compound, to which we assigned the structure of the amide acetal **11** on the basis of NMR data. The new structure was then verified by X-ray crystallography (Fig. 1).‡
‡Crystal data. C_10_H_12_N_4_O_5_·H_2_O, *M* = 286.25, orthorhombic, *a* = 7.4674(2), *b* = 10.9703(3), *c* = 13.9309(4) Å, *U* = 1141.21(5) Å^3^, *T* = 180 K, space group *P*2_1_2_1_2_1_ (no. 19), *Z* = 4, 13 491 reflections measured, 1996 unique (*R*_int_ = 0.030), which were used in all calculations. The final w*R*(*F* ^2^) was 0.069 (all data). At first glance, the structure of **11** may appear surprising, but by drawing an analogy to the reaction of ribose aminooxazoline **1** and cyanoacetylene **2** (Scheme 1), a cascade of reactions leading to **11** from **1** and dicyanoacetylene **5** can easily be envisaged (Scheme 2). Thus, we propose that conjugate addition of **1** to **5** gives an intermediate **6**, which undergoes spontaneous 6-*exo-dig* addition to form the anhydronucleoside **7** – the 6-cyano-analogue of anhydro-α-cytidine **3**. Again by comparison to the reaction of ribose aminooxazoline **1** and cyanoacetylene **2**, the next step in the cascade leading from **1** and dicyanoacetylene **5** is expected to be the hydrolysis of the anhydronucleoside **7** induced by an increase in the pH of the system due to protonation of the free base form of the anhydronucleoside. Indeed, the pH of the reaction mixture after the addition of dicyanoacetylene **5** increased to 7.8, conditions that should be conducive to the hydrolytic ring-opening of anhydronucleoside **7** to the lactim **8** and thence, after tautomerisation, 6-cyano-α-cytidine derivative **9**. However, this latter compound was not isolated and we think it immediately underwent an intramolecular Pinner reaction,^8^ presumably with the 2′-hydroxyl group, to give an imidate **10** which, in turn, underwent addition, presumably with the 3′-hydroxyl group, to give the amide acetal **11**. The yield of **11** based on **1** was initially quite poor, but was increased to 32% by inclusion of phosphate buffer (6.9 eq. **5**, pH = 6.9, 100 mM phosphate buffer). This buffering effect suggests that the hydrolysis of anhydronucleoside **7** occurs at a lower pH than the hydrolysis of the des-cyano analogue **3**. Inclusion of more equivalents of **5** was dentrimental to the obtention of **11** and products containing two or three dicyanoethylene groups were additionally observed. Although the intermediates **7** and **9** in this cascade of reactions were not isolated, reaction time course ^1^H NMR spectroscopic observations were in agreement with our proposed reaction scheme. In the ^1^H NMR spectra taken 10 min and 1 h from the beginning of the reaction (Fig. 2a and b), we observed a triplet peak which we assign to the 2′-proton of anhydronucleoside **7** at *δ* 5.66 (*J* = 5.4 Hz) in good agreement with data of other anhydronucleoside derivatives.^2,3^ Evidence for the intermediacy of the hydrolysis product **9** was provided by a multiplet at *δ* 4.35 for the 2′- and 3′-protons (the 2′-proton signal being upfield relative to that of the anhydronucleoside precursor **7**), along with a doublet at *δ* 6.03 (*J* = 3.8 Hz) for the 1′-proton. Although the initial ^1^H NMR spectra were quite complex, the intermediates were eventually consumed leaving almost exclusively starting material **1** and the product **11** (Fig. 2c). The ^1^H NMR spectrum of pure **11** confirmed its presence in the crude reaction products (Fig. 2d).

**Fig. 1:**
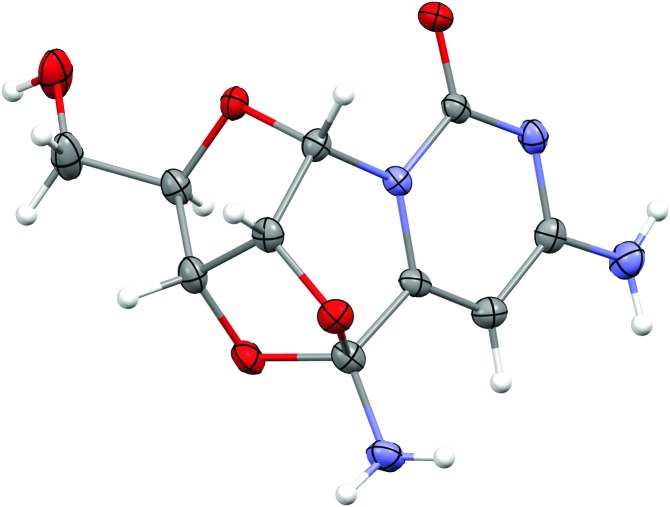
Crystal structure of the tetracyclic compound **11**.

**Scheme 2:**
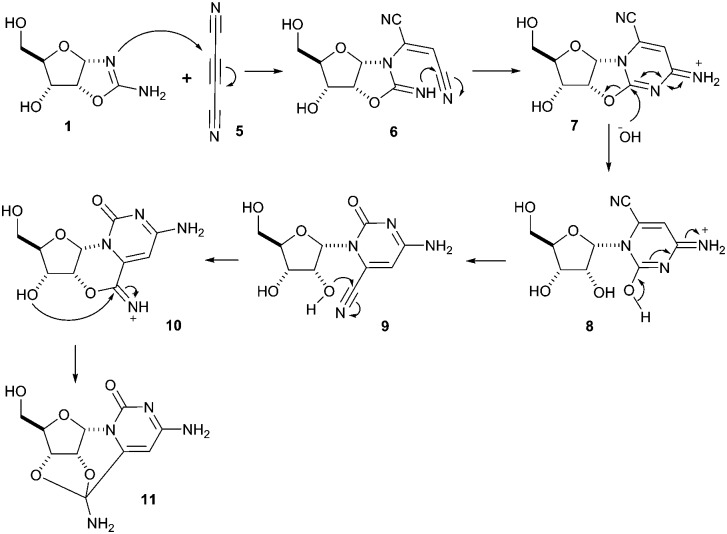
Reaction of ribose aminooxazoline **1** with dicyanoacetylene **5**.

**Fig. 2:**
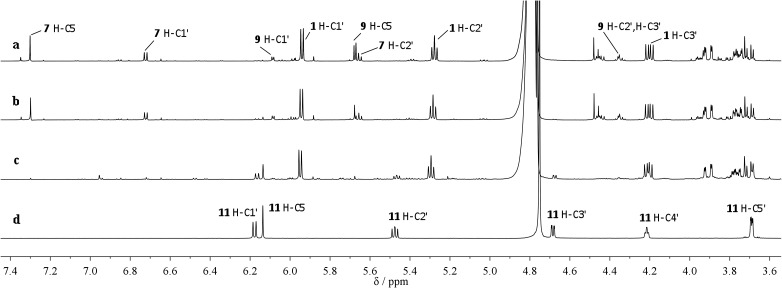
Time course ^1^H NMR spectroscopy of the reaction of **1** with **5** (a) after 10 min (b) after 1 h (c) after 22 h (d) pure product **11**.

Given the unusual structure of **11**, where a nitrile group is effectively masked as an amide acetal function, and its potential relevance to prebiotic chemistry, we were keen to develop a synthesis which did not involve the starting material **5**, because use of the latter would be hazardous on a large scale. The idea for our alternative synthesis of **11** was to introduce the nitrile group on the 6-position of α-cytidine **4** (Scheme 3). A similar synthesis was followed in 1978 for the cyanation of β-cytidine through a 5-bromo intermediate.^9^ The starting material, α-cytidine **4**, was easily synthesized following the steps shown in Scheme 1.^2^ In the first step of the subsequent conventional synthesis of **11**, α-cytidine **4** was selectively acetylated on the amino group, giving the acetyl derivative **12**, by refluxing in a methanolic solution containing acetic anhydride.^10^ The amide **12** was then perbenzoylated in a second step with benzoyl chloride yielding the tribenzoyl ester, which was subsequently deacetylated to afford the desired free amine **13**. The latter was used for the reaction with bromine in acetic acid giving the protected 5-bromo-α-cytidine **14**. The key step of this reaction sequence proved to be the cyanation of **14**. It was found that a slower reaction in a diluted mixture having no excess of sodium cyanide provided the desired product **15** in the best yield, while more concentrated reaction mixtures gave rise to many side products, thus lowering the yield of **15**. While cleaving the benzoyl esters with sodium methoxide, 6-cyano-α-cytidine **9** was formed, but was not isolated, as the cyano group reacted further under these conditions to give the amide acetal product **11** identical in all respects with material synthesised under prebiotic conditions.

**Scheme 3:**
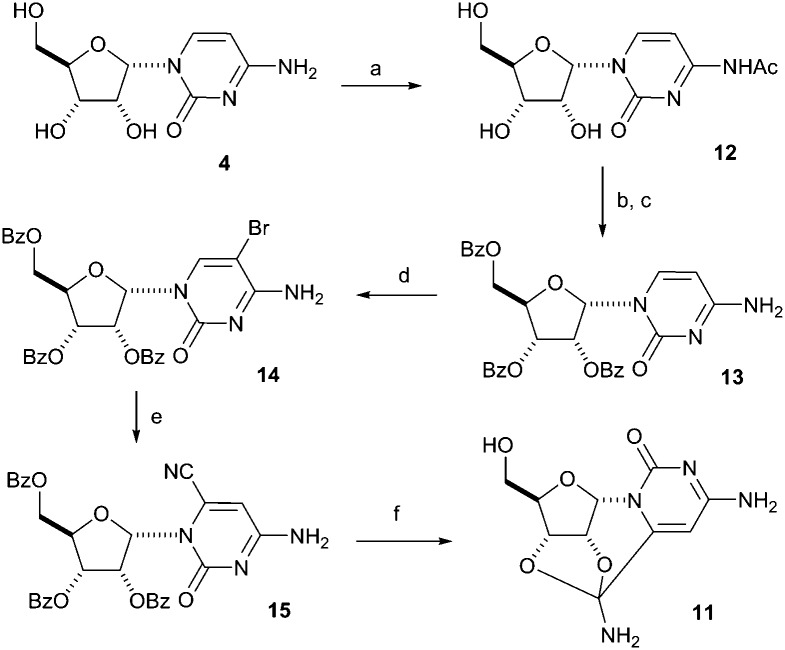
Synthesis of amide acetal **11** following a 6 step synthetic sequence. (a) Ac_2_O, MeOH dry, reflux, 1 h (yield 92%), (b) BzCl, py, rt, overnight, (c) MeOH dry, AcOH, reflux, overnight (yield 74%), (d) AcOH, py, Br_2_, rt, overnight (yield 76%), (e) DMF, NaCN, rt, 2 d (yield 62%), (f) NaOMe, MeOH dry, rt. 2 h (yield 99%).

In conclusion, the potentially prebiotic reaction of ribose aminooxazoline **1** with dicyanoacetylene **5** was studied and found to afford the amide acetal **11**, which was also synthesised by more conventional means. Coincidentally, both the procedures had the same total yield (32%), but the second one can be used more efficiently and safely at larger scales. Even though nucleosides having the α-anomeric configuration are not found in natural nucleic acids, they constitute important intermediates in the prebiotic synthesis of the nucleotides^3^ and also some of them have been found to exhibit pronounced antimetabolic activities – whether this is true of **11** has not been established.^11^

This work was supported by the Medical Research Council (No. MC_UP_A024_1009), and a grant from the Simons Foundation (No. 290362 to J. D. S.).
