# Supplementary material for: A new and potentially prebiotic α-cytidine derivative
Source: Chem Commun (Camb). 2017 Feb 23;53(23):3327–9. doi: 10.1039/c7cc00693d (PMC5510716; doi:10.1039/c7cc00693d)
Supplement: Supplementary file 1 [file CC-053-C7CC00693D-s001.pdf]

Electronic Supplementary Material (ESI) for ChemComm.
This journal is © The Royal Society of Chemistry 2017

Supporting Information

A new and potentially prebiotic α-cytidine derivative

Maria Tsanakopoulou,[a] Jianfeng Xu,[a] Andrew D. Bond[b] and John D. Sutherland*[a]

[a] *MRC Laboratory of Molecular Biology, Francis Crick Avenue, Cambridge Biomedical Campus, CB2 0QH, UK. Email: johns@mrc-lmb.cam.ac.uk*
[b] *Department of Chemistry, University of Cambridge, Lensfield Road, CB2 1EW, UK.*

Table of contents

1. General methods S1
2. Synthesis of dicyanoacetylene **5** S1
3. Reaction of ribose aminooxazoline **1** with dicyanoacetylene **5** S2
4. Synthesis of *N*-acetyl-α-cytidine **12** S5
5. Synthesis of 2′,3′,5′-tribenzoyl-α-cytidine **13** S8
6. Synthesis of 2′,3′,5′-tribenzoyl-5-bromo-α-cytidine **14** S11
7. Synthesis of 2′,3′,5′-tribenzoyl-6-cyano-α-cytidine **15** S14
8. Synthesis of amide acetal **11** S17

1. General methods

All reagents and solvents were purchased from Sigma-Aldrich and Acros Organics and were used without further purification. A *Mettler Toledo* SevenEasy pH Meter S20 with a *ThermoFisher Scientific* Orion 8103BN Ross combination semi-micro pH electrode was used to measure and adjust the pH. A *Varian* ProStar HPLC System was used for the reverse phase high-pressure liquid chromatography (RP-HPLC) linked with an *Atlantis* T3 C18 Prep Column OBD 10 µm (19×250 mm). A *Grace* Reveleris Prep purification system was used for direct phase chromatography with Reveleris Flash silica cartridges. ^{1}H and ^{13}C NMR spectra were acquired using a *Bruker* Ultrashield 400 Plus operating at 400.1 MHz and 100.6 MHz respectively. Samples consisting of H_2O/D_2O mixtures were analysed using HOD suppression to collect ^{1}H NMR spectroscopy data. Chemical shifts (δ) are shown in ppm. The yields of conversion were determined by relative integrations of the signals in the ^{1}H NMR spectrum. Coupling constants (*J*) are given in Hertz and the notations s, d, m represent the multiplicities singlet, doublet and multiplet. Mass spectra were recorded with an *Agilent Technologies* 6130 Quadrupole LC-MS using positive and negative Electron Spray Ionisation. The accurate mass spectra were recorded with a *Waters* Vion IMS QToF Ion Mobility Quadrupole Time-of-flight Mass spectrometer using positive ESI.

2. Synthesis of dicyanoacetylene 5

Dicyanoacetylene **5** was synthesised following a slightly modified literature method.[1] Acetylenedicarboxamide (2.0 g, 17.9 mmol) was mixed with dried and calcined sand (15.0 g) and phosphorus pentoxide (15.0 g, 52.8 mmol) using a

mortar and pestle. The solid mixture was transferred into a 250 mL round-bottom flask and was heated at 215°C into preheated high temperature silicone oil. An inlet and outlet allowed argon to flow over the mixture, thus facilitating the transfer of the gaseous product into three consecutive connected flasks, which were cooled by acetone-dry ice baths for the collection of the distilled product. The argon flow was then led through a calcium chloride tube and finally through a trap containing sodium hypochlorite solution 6%. The reaction was completed after 15-20 min of heating and the flasks, containing 300 mg of the white crystalline solid **5** (yield 22%), were capped with glass stoppers, sealed with film and stored in the freezer at -30°C for several days. The ^{13}C NMR spectrum was found to be in agreement with the one reported in the literature.[2]

3. Reaction of ribose aminooxazoline 1 with dicyanoacetylene 5

Ribose aminooxazoline **1** (20 mg, 0.115 mmol) and NaH_2PO_4 (16 mg, 0.100 mmol) were dissolved in water (0.5 mL, containing 10% D_2O) and the pH of the mixture was adjusted to 6.9 with 1M HCl. Dicyanoacetylene **5** (60 mg, 0.789 mmol) was dissolved in water (0.5 mL, containing 10% D_2O) and was immediately added to the reaction. The mixture was transferred into an NMR tube and was monitored by ^1H-NMR. After 3 d at rt, the ^1H-NMR spectrum showed that the reaction was not proceeding any further and that the final product **11** was formed in 32% yield. The mixture was separated by RP-HPLC using a water-acetonitrile gradient to give, after lyophilisation, the white crystalline amide acetal **11** (9 mg, 30%). ^1H NMR (400 MHz, D_2O) δ 6.13 (d, *J* = 6.0 Hz, 1H, H1′), 6.09 (s, 1H, H5), 5.48 – 5.36 (m, 1H, H2′), 4.64 (dd, *J* = 5.0, 1.2 Hz, 1H, H3′), 4.17 (t, *J* = 2.9 Hz, 1H, H4′), 3.68 – 3.59 (m, 2H, H5′, H5′′); ^{13}C NMR (101 MHz, D_2O) δ 166.0 (C4), 158.3 (C=O, C2), 152.3 (C6), 107.4 (C-NH_2), 90.3 (C5), 83.9 (C4′), 80.2 (C1′), 79.1 (C3′), 77.9 (C2′), 60.9 (C5′); ESI-LCMS (pos. m/z) 269.1 $[M+H]^+$, 291.0 $[M+Na]^+$; ESI-HRMS (pos. m/z) $[M+H]^+$ calcd for $C_{10}H_{13}N_4O_5$ 269.0886; found 269.0876.

Entry	Eq. of 5	pH	Phosphate buffer	Yield of 11
**1**	5.7	7.0	-	9%
**2**	5.7	7.0	0.1M	21%
**3**	6.4	6.5	0.1M	27%
**4**	6.9	6.9	0.1M	32%
**5**	13.7	6.9	0.1M	30%

Table 1 Conditions and yield for the reaction of ribose aminooxazoline **1** with dicyanoacetylene **5**

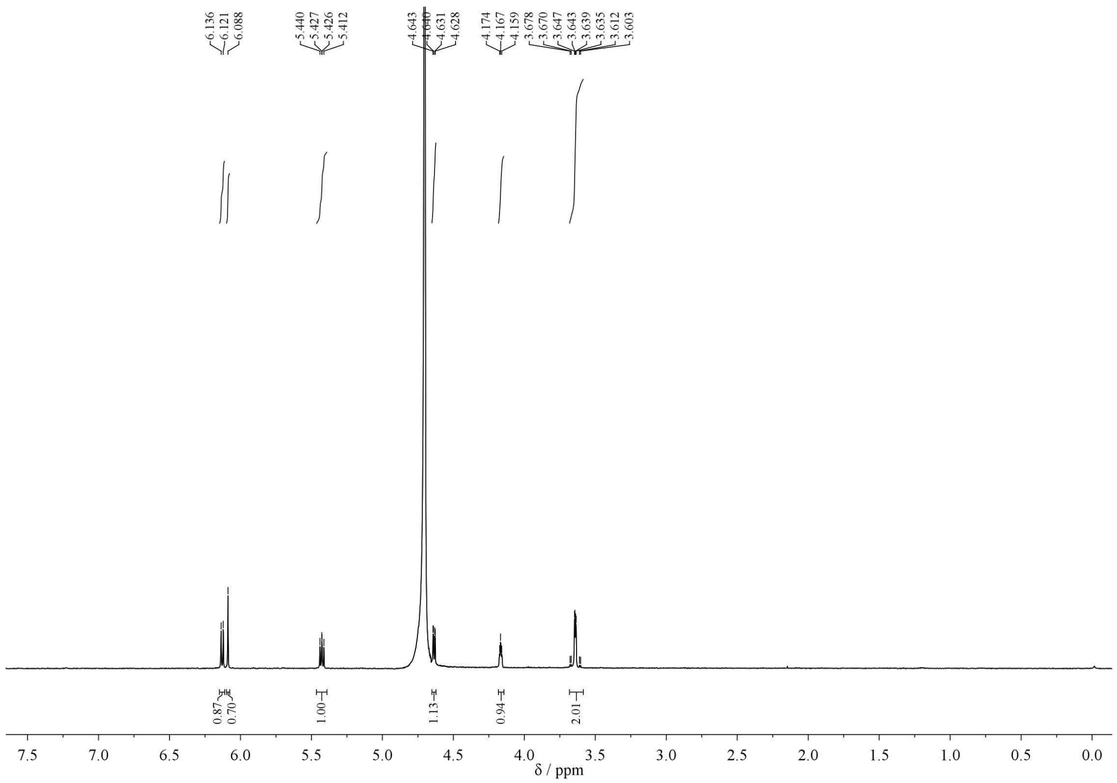

Figure S1. ^{1}H NMR spectrum of amide acetal **11** in D_2O.

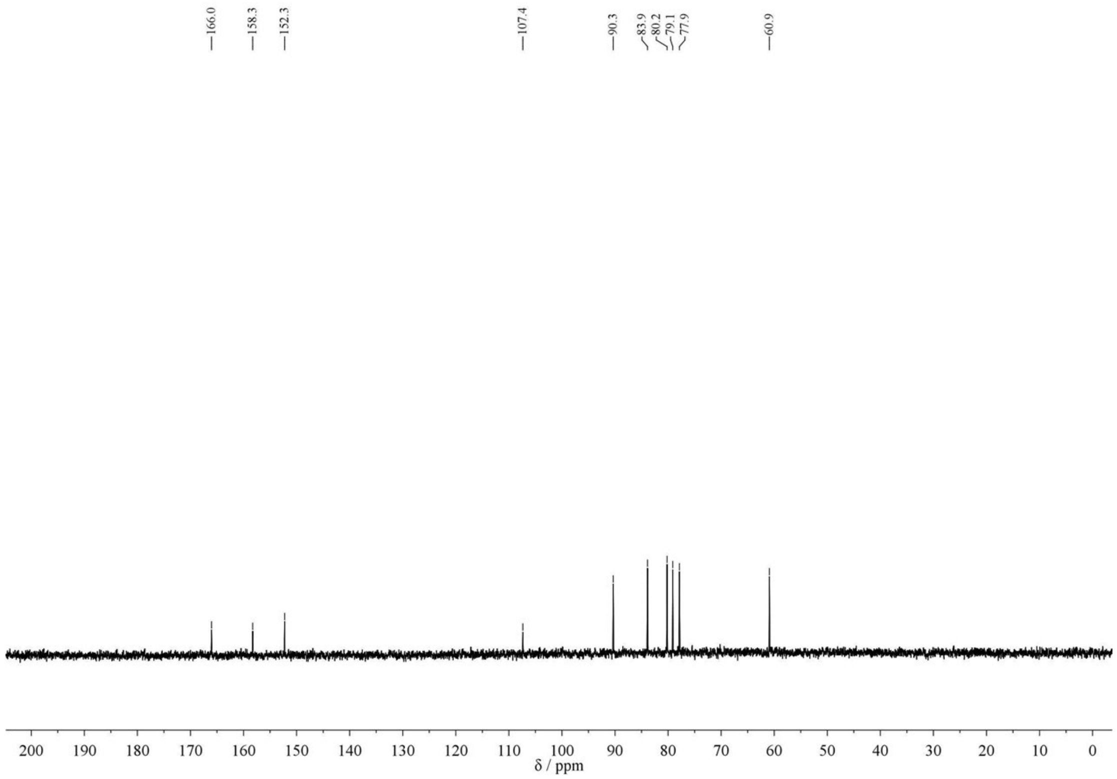

Figure S2. ^{13}C NMR spectrum of amide acetal **11** in D_2O.

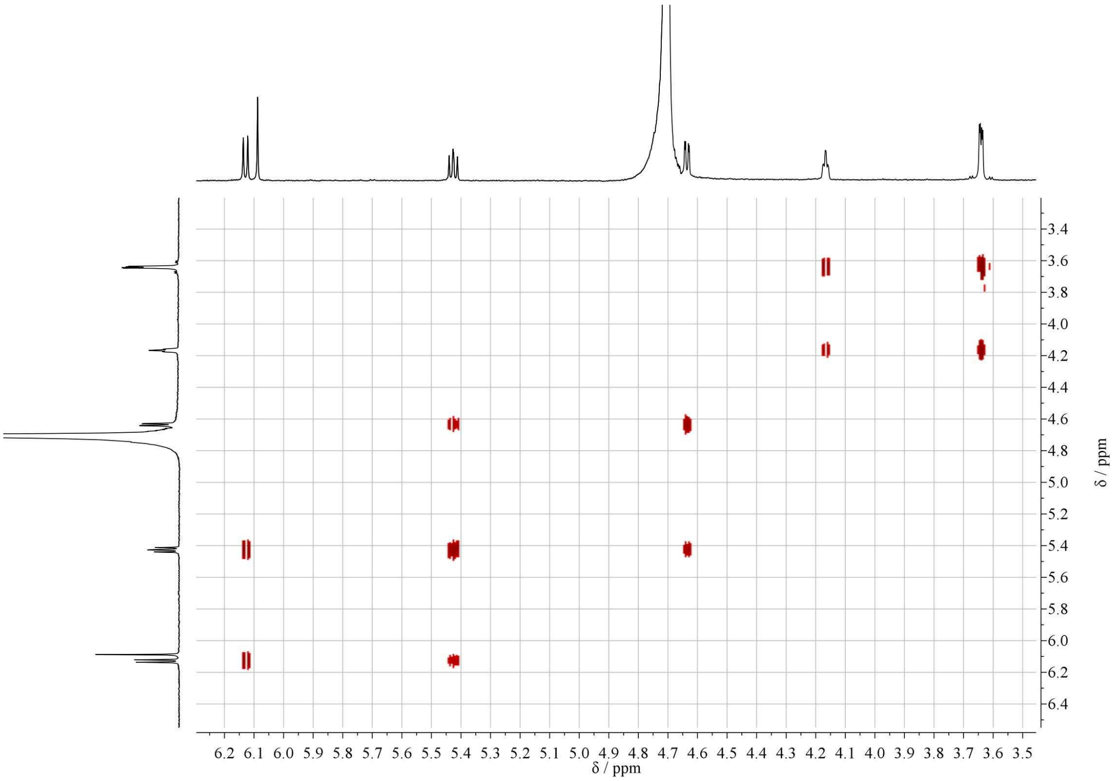

Figure S3. COSY NMR spectrum of amide acetal **11** in D_2O.

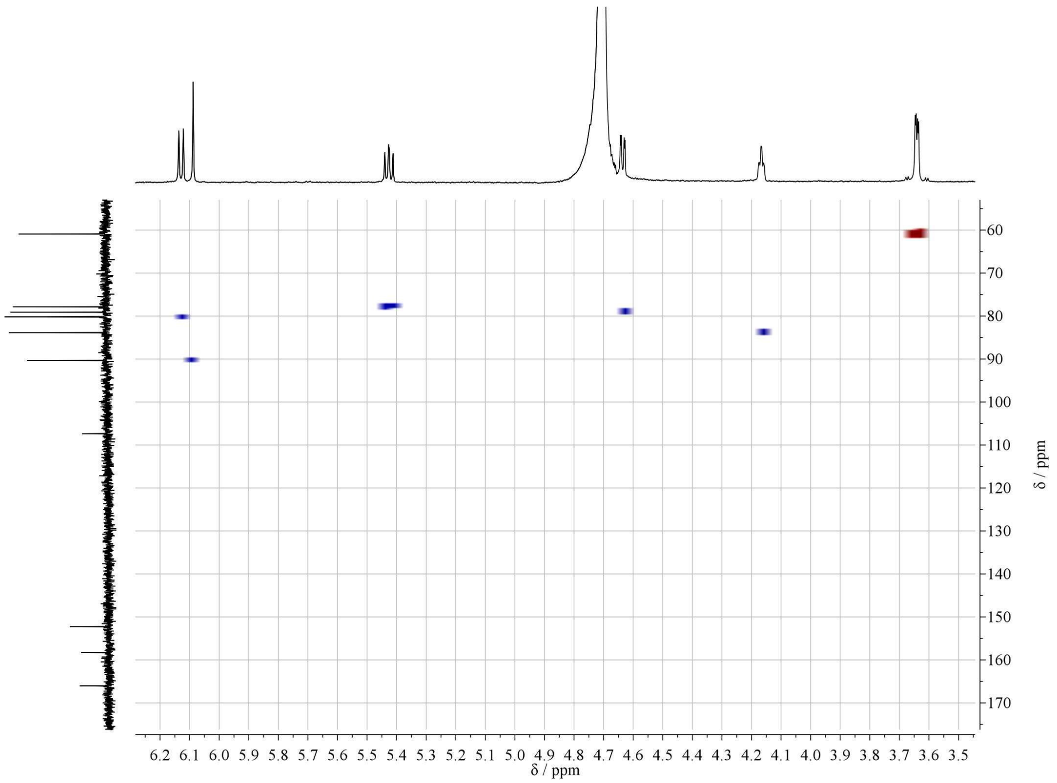

Figure S4. HSQC NMR spectrum of amide acetal **11** in D_2O.

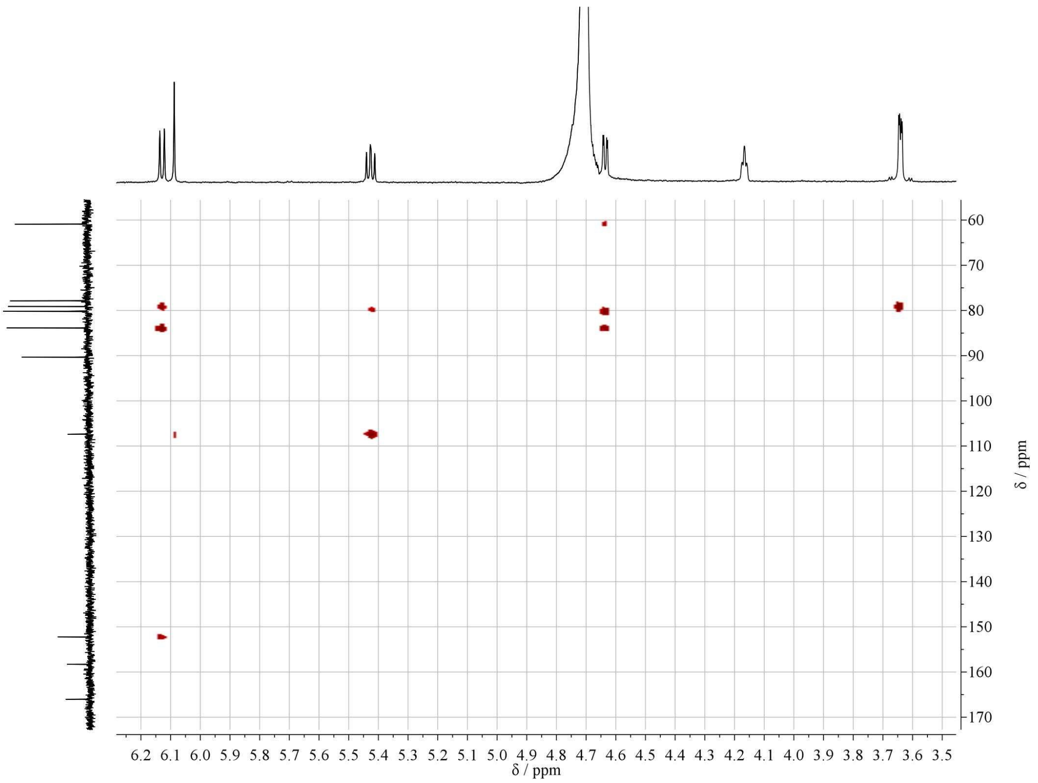

Figure S5. HMBC NMR spectrum of amide acetal **11** in D_2O.

4. Synthesis of *N*-acetyl-α-cytidine 12

α-Cytidine **4**[3] (167 mg, 0.69 mmol) was suspended in dry methanol (6 mL) and then acetic anhydride (0.67 mL, 7.10 mmol) was added. The mixture was refluxed for 1.5 h, until TLC indicated the consumption of the starting material. The mixture was evaporated and then suspended in diethylether, filtered and dried under vacuum. The white solid was pure enough in order to be used for the next step (180 mg, yield 92%), although an analytically pure sample of **12** was obtained by RP-HPLC using a water-acetonitrile gradient. ^{1}H NMR (400 MHz, D_2O) δ 8.10 (d, *J* = 7.5 Hz, 1H, H6), 7.30 (d, *J* = 7.6 Hz, 1H, H5), 6.11 (d, *J* = 3.8 Hz, 1H, H1′), 4.50 (t, *J* = 4.1 Hz, 1H, H2′), 4.29 (dd, *J* = 8.0, 4.4 Hz, 1H, H3′), 4.24 (ddd, *J* = 8.0, 4.6, 2.4 Hz, 1H, H4′), 3.89 (dd, *J* = 12.7, 2.4 Hz, 1H, H5′), 3.68 (dd, *J* = 12.7, 4.6 Hz, 1H, H5″), 2.18 (s, 3H, Me); ^{13}C NMR (101 MHz, D_2O) δ 174.0 (C=O), 162.6 (C4), 156.8 (C=O, C2), 146.6 (C6), 97.2 (C5), 88.0 (C1′), 83.0 (C4′), 70.4 (C2′), 70.1 (C3′), 60.7 (C5′), 24.0 (Me); ESI-LCMS (pos. m/z) 286.1 $[M+H]^+$, 308.1 $[M+Na]^+$; (neg. m/z) 284.0 $[M-H]^-$; ESI-HRMS (pos. m/z) $[M+Na]^+$ calcd for $C_{11}H_{15}N_3NaO_6$ 308.0859; found 308.0844.

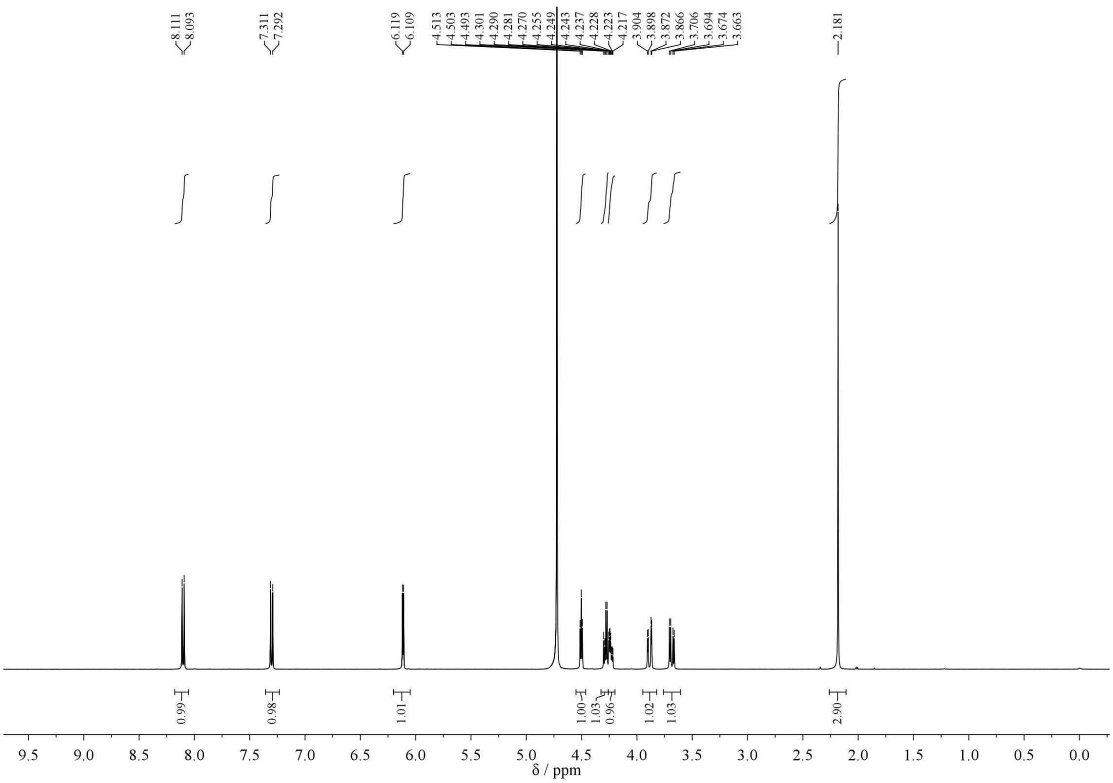

Figure S6. ^{1}H NMR spectrum of *N*-acetyl-α-cytidine **12** in D_2O.

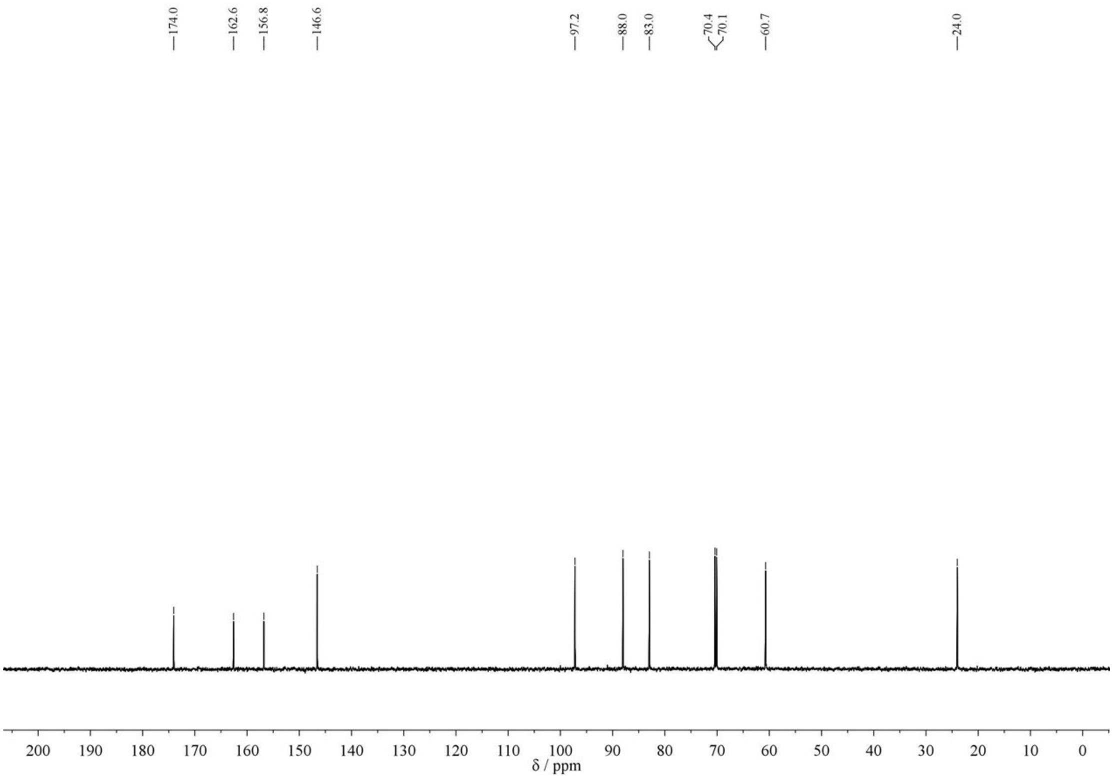

Figure S7. ^{13}C NMR spectrum of *N*-acetyl-α-cytidine **12** in D_2O.

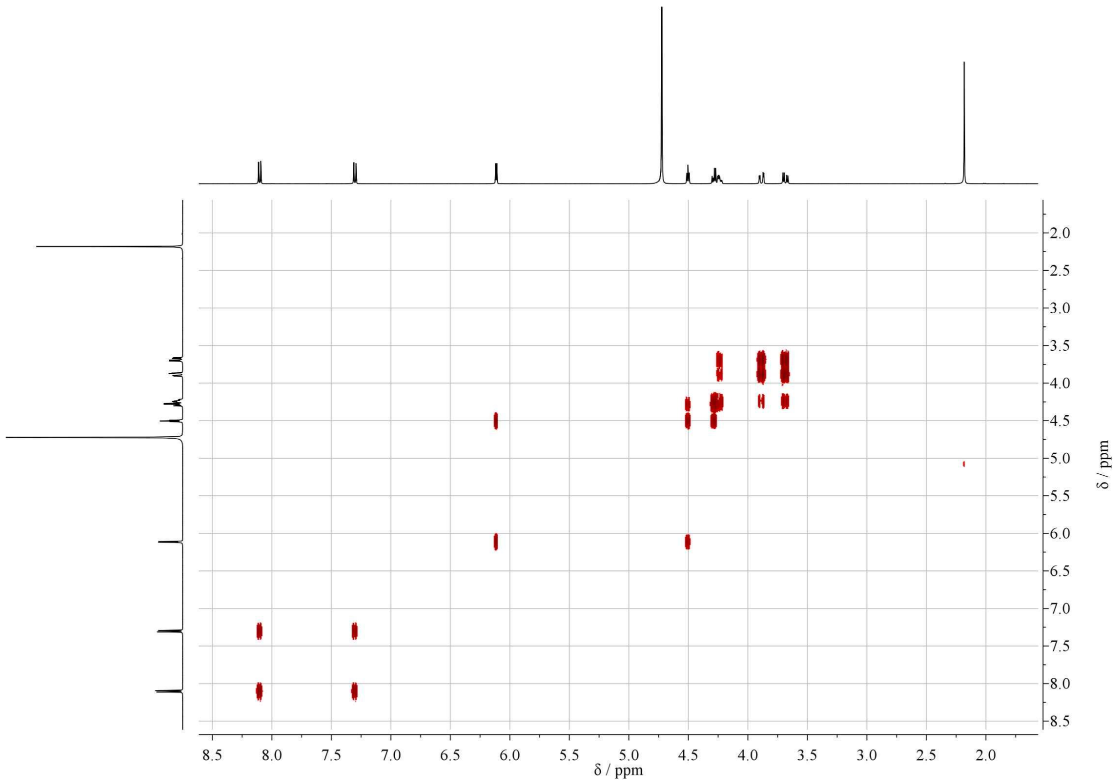

Figure S8. COSY NMR spectrum of *N*-acetyl-α-cytidine **12** in D_2O.

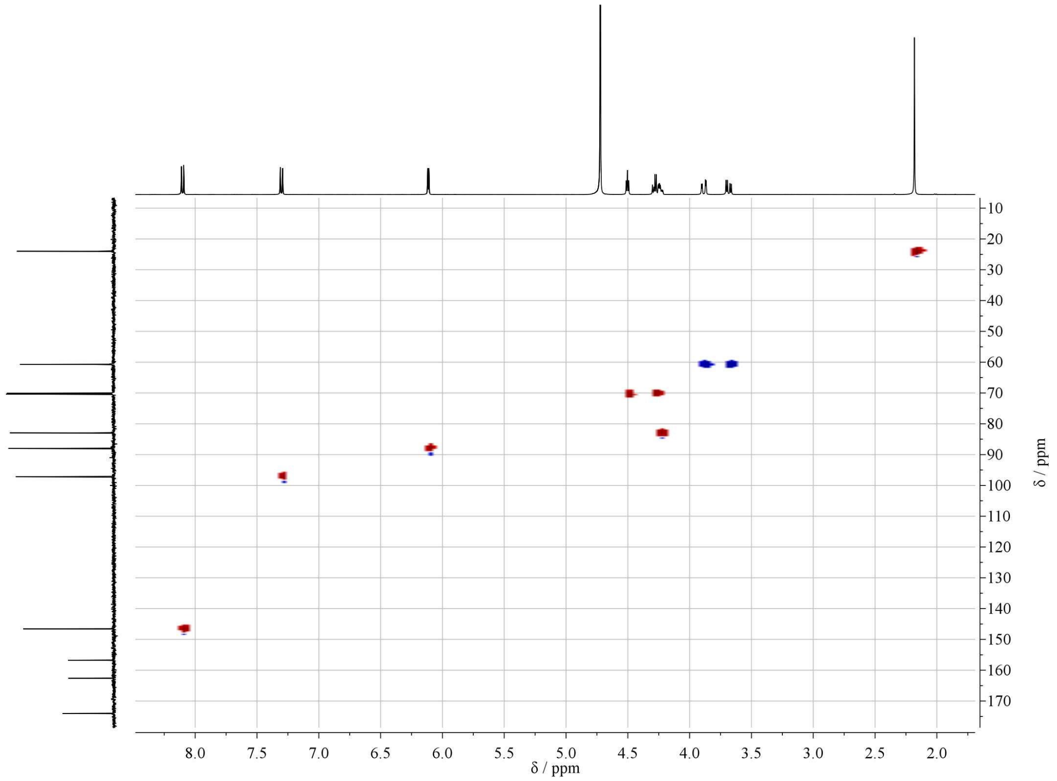

Figure S9. HSQC NMR spectrum of *N*-acetyl-α-cytidine **12** in D_2O.

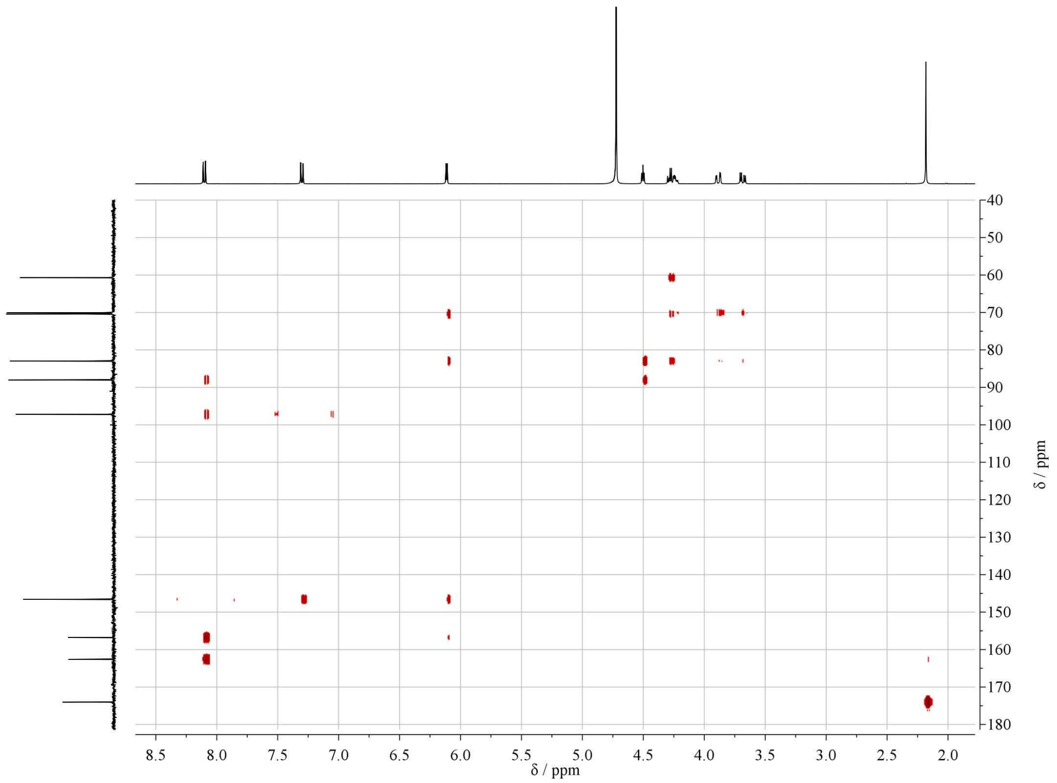

Figure S10. HMBC NMR spectrum of *N*-acetyl-α-cytidine **12** in D_2O.

5. Synthesis of 2′,3′,5′-tribenzoyl-α-cytidine 13

N-Acetyl-α-cytidine **12** (100 mg, 0.35 mmol) was dissolved in dry pyridine (10 mL) under nitrogen. Benzoyl chloride (0.20 mL, 1.75 mmol) was added and the mixture was stirred at rt overnight. Bulk pyridine was evaporated and then residual pyridine was co-evaporated with methanol. The crude product was dried under vacuum and used for the next step immediately. For this purpose, it was dissolved in dry methanol (15 mL), acetic acid was added (0.12 mL, 2.10 mmol) and the mixture was refluxed overnight. The solvent was removed by evaporation and the residue was dried under vacuum, separated by flash column chromatography using solvent mixtures of DCM:MeOH increasing the polarity from 99:1 to 90:10 to give, after evaporation, 144 mg of the pure product **13** (yield 74%, over two steps). ^{1}H NMR (400 MHz, $CDCl_3$) δ 8.09 – 7.91 (m, 3H, Bz), 7.77 – 7.65 (m, 4H, Bz), 7.62 (d, *J* = 7.5 Hz, 1H, H6), 7.54 – 7.44 (m, 1H, Bz), 7.41 (t, *J* = 7.5 Hz, 1H, Bz), 7.35 (dt, *J* = 7.7, 2.4 Hz, 3H, Bz), 7.20 (t, *J* = 7.5 Hz, 4H, Bz), 6.58 (d, *J* = 4.4 Hz, 1H, H1′), 6.07 (t, *J* = 4.6 Hz, 1H, H2′), 5.83 (dd, *J* = 6.2, 5.0 Hz, 1H, H3′), 5.76 (d, *J* = 7.5 Hz, 1H, H5), 4.84 – 4.76 (m, 1H, H4′), 4.66 (dd, *J* = 12.1, 3.5 Hz, 1H, H5′), 4.50 (dd, *J* = 12.1, 4.2 Hz, 1H, H5″); ^{13}C NMR (101 MHz, $CDCl_3$) δ 166.1 (C=O, Bz5′), 165.9 (C4), 165.1 (C=O, Bz3′), 164.5 (C=O, Bz2′), 155.5 (C=O, C2), 141.0 (C6), 133.6, 133.5, 133.4, 129.7, 129.7, 129.6, 129.2, 128.7, 128.6, 128.5, 128.4, 94.3 (C5), 85.5 (C1′), 79.6 (C4′), 71.9 (C3′), 71.2 (C2′), 63.8 (C5′); ESI-LCMS (pos. m/z) 556.0 $[M+H]^+$, 578.0 $[M+Na]^+$; ESI-HRMS (pos. m/z) $[M+Na]^+$ calcd for $C_{30}H_{25}N_3NaO_8$ 578.1539; found 578.1526.

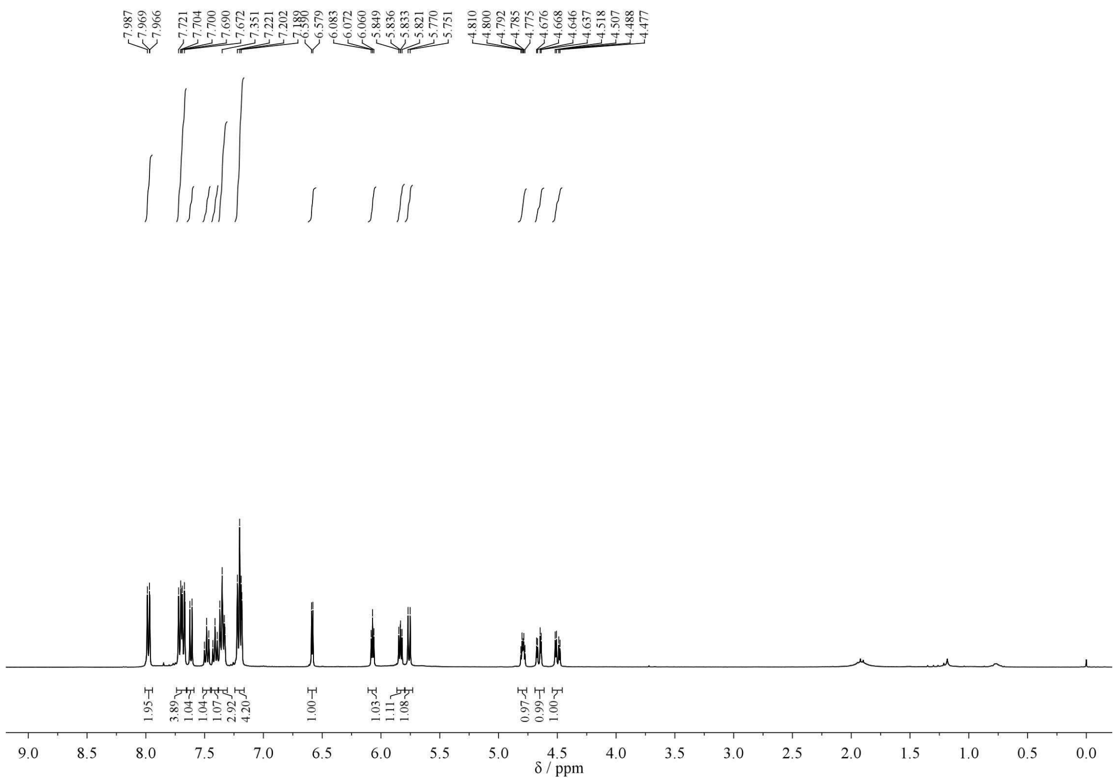

Figure S11. ^{1}H NMR spectrum of 2′,3′,5′-tribenzoyl-α-cytidine **13** in $CDCl_3$.

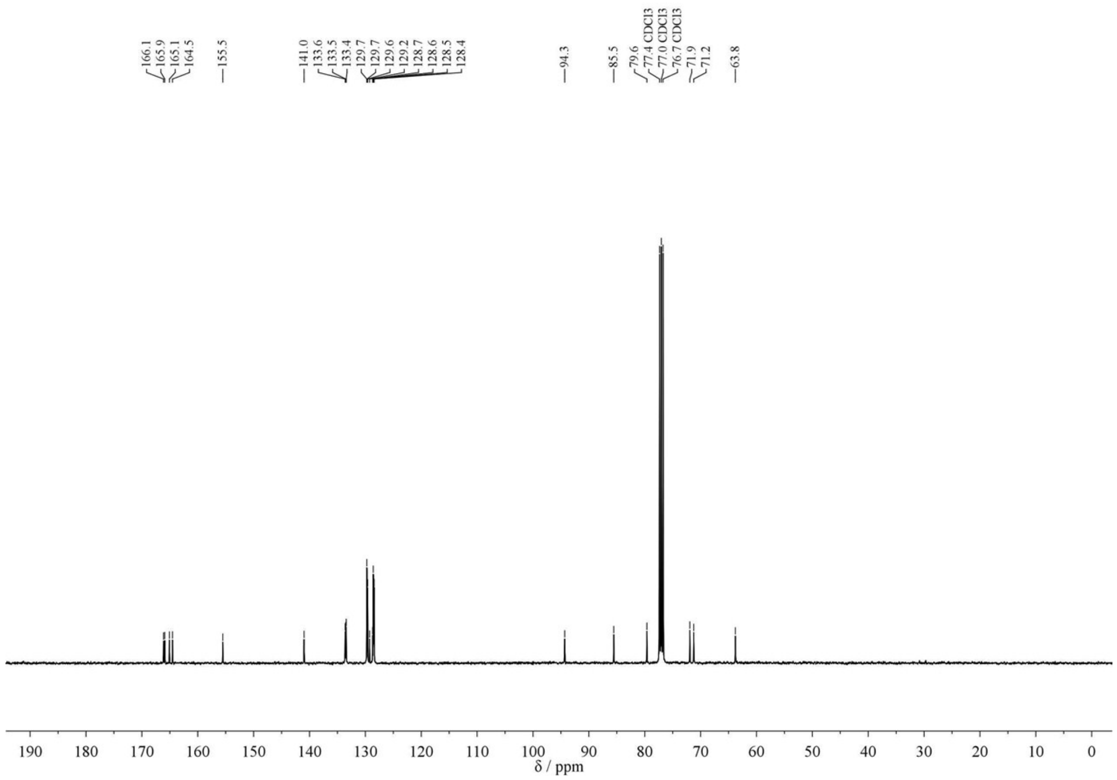

Figure S12. ^{13}C NMR spectrum of 2′,3′,5′-tribenzoyl-α-cytidine **13** in $CDCl_3$.

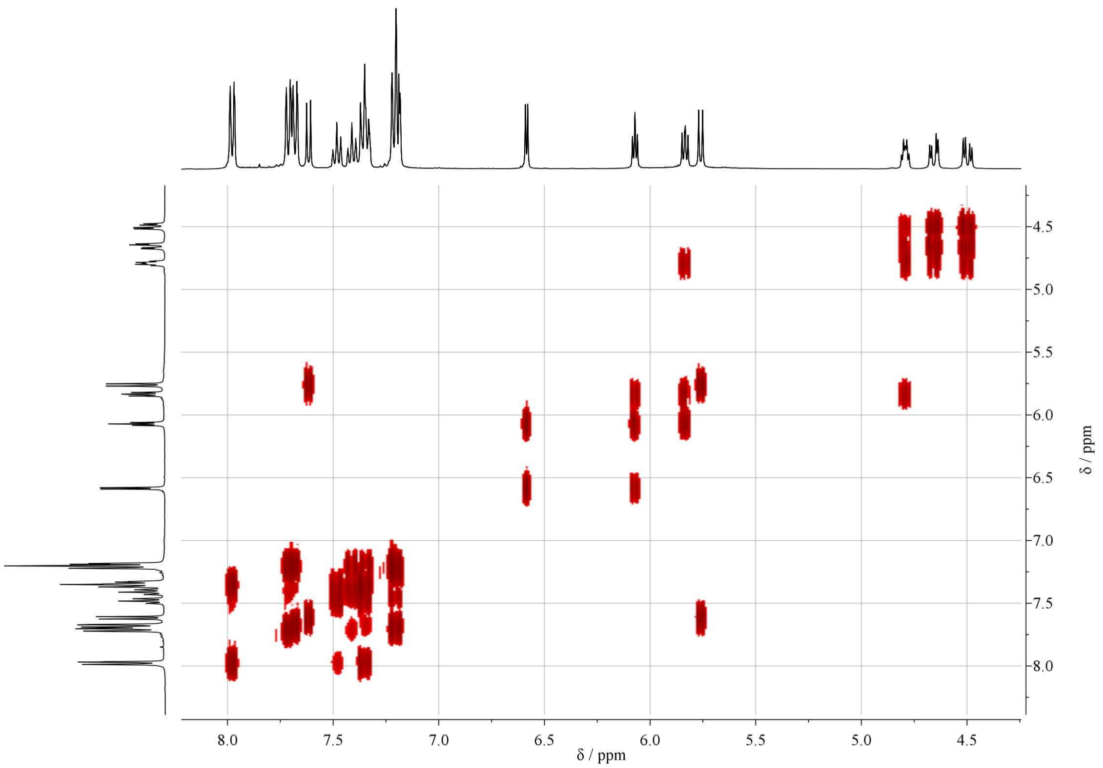

Figure S13. COSY NMR spectrum of 2′,3′,5′-tribenzoyl-α-cytidine **13** in $CDCl_3$.

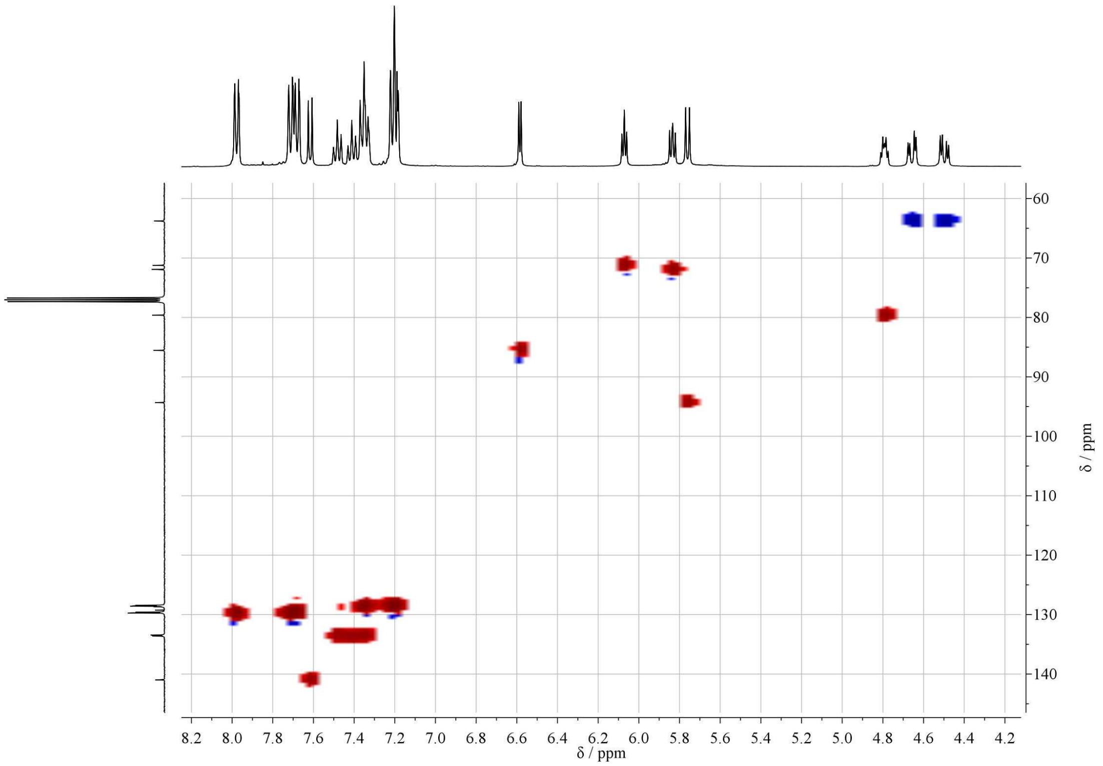

Figure S14. HSQC NMR spectrum of 2′,3′,5′-tribenzoyl-α-cytidine **13** in $CDCl_3$.

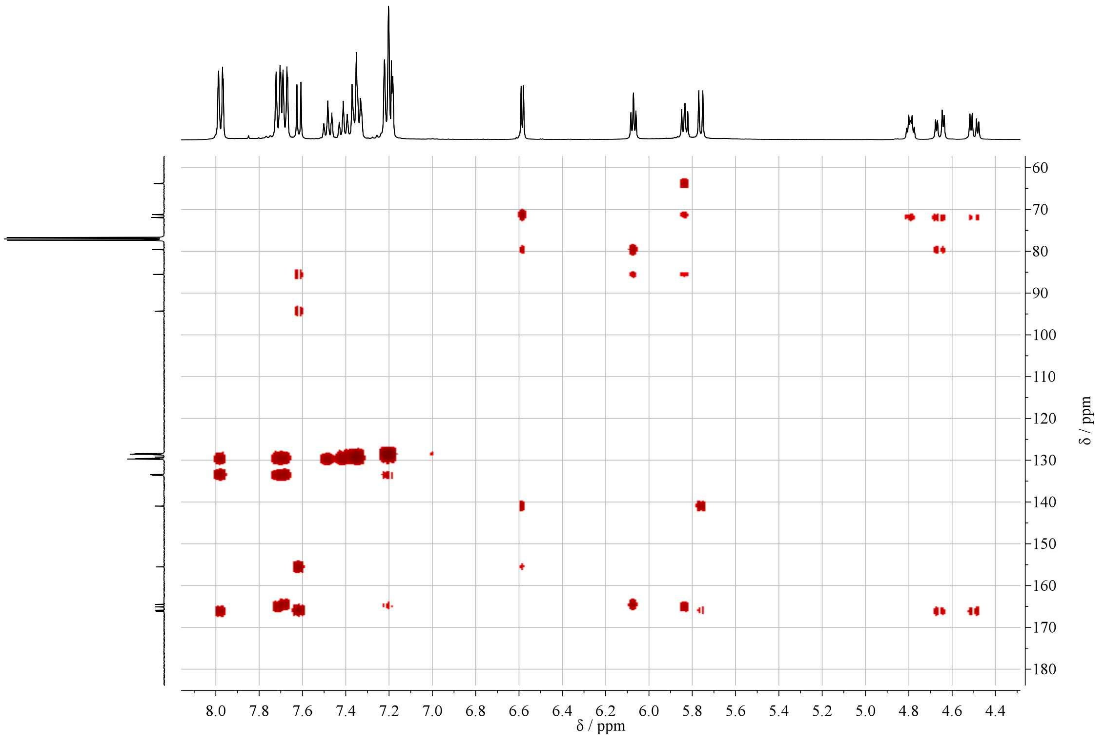

Figure S15. HMBC NMR spectrum of 2′,3′,5′-tribenzoyl-α-cytidine **13** in $CDCl_3$.

6. Synthesis of 2′,3′,5′-tribenzoyl-5-bromo-α-cytidine 14

2′,3′,5′-Tribenzoyl-α-cytidine **13** (123 mg, 0.22 mmol) was dissolved in dry pyridine (1 mL) and acetic acid (1 mL). Bromine was added (0.015 mL, 0.33 mmol) at rt and the reaction mixture was stirred overnight. The light yellow solution was evaporated, co-evaporated with ethanol and dried under vacuum. The residual oil was separated by Grace column chromatography using solvent mixtures of DCM:MeOH increasing the polarity from 99:1 to 90:10 to give after evaporation 103 mg of the pure product **14** (yield 75%). ^1H NMR (400 MHz, $CDCl_3$) δ 8.93 (brs, 1H, NH), 8.10 – 7.92 (m, 2H, Bz), 7.85 (s, 1H, H6), 7.78 – 7.72 (m, 2H, Bz), 7.72 – 7.68 (m, 2H, Bz), 7.52 – 7.46 (m, 1H, Bz), 7.46 – 7.40 (m, 1H, Bz), 7.39 – 7.31 (m, 3H, Bz), 7.24 (td, *J* = 7.8, 3.9 Hz, 4H, Bz), 6.62 (d, *J* = 4.7 Hz, 1H, H1′), 6.06 (t, *J* = 4.9 Hz, 1H, H2′), 5.88 (t, *J* = 5.1 Hz, 1H, H3′), 5.72 (brs, 1H, NH), 4.94 – 4.83 (m, 1H, H4′), 4.65 (dd, *J* = 12.2, 3.6 Hz, 1H, H5′), 4.51 (dd, *J* = 12.2, 4.0 Hz, 1H, H5″); ^{13}C NMR (101 MHz, $CDCl_3$) δ 166.1 (C=O, Bz5′), 165.0 (C=O, Bz3′), 164.3 (C=O, Bz2′), 162.2 (C=O, C4), 154.0 (C4), 141.9 (C6), 133.7, 133.6, 133.5, 129.7, 129.6, 129.1, 128.7, 128.6, 128.6, 128.4, 87.0 (C5) 85.5 (C1′), 80.4 (C4′), 72.0 (C3′), 71.1 (C2′), 63.8 (C1′); ESI-LCMS (pos. m/z) 634.0, 636.0 $[M+H]^+$, 656.0, 658.0 $[M+Na]^+$; ESI-HRMS (pos. m/z) $[M+Na]^+$ calcd for $C_{30}H_{24}BrN_3NaO_8$ 656.0645; found 656.0626.

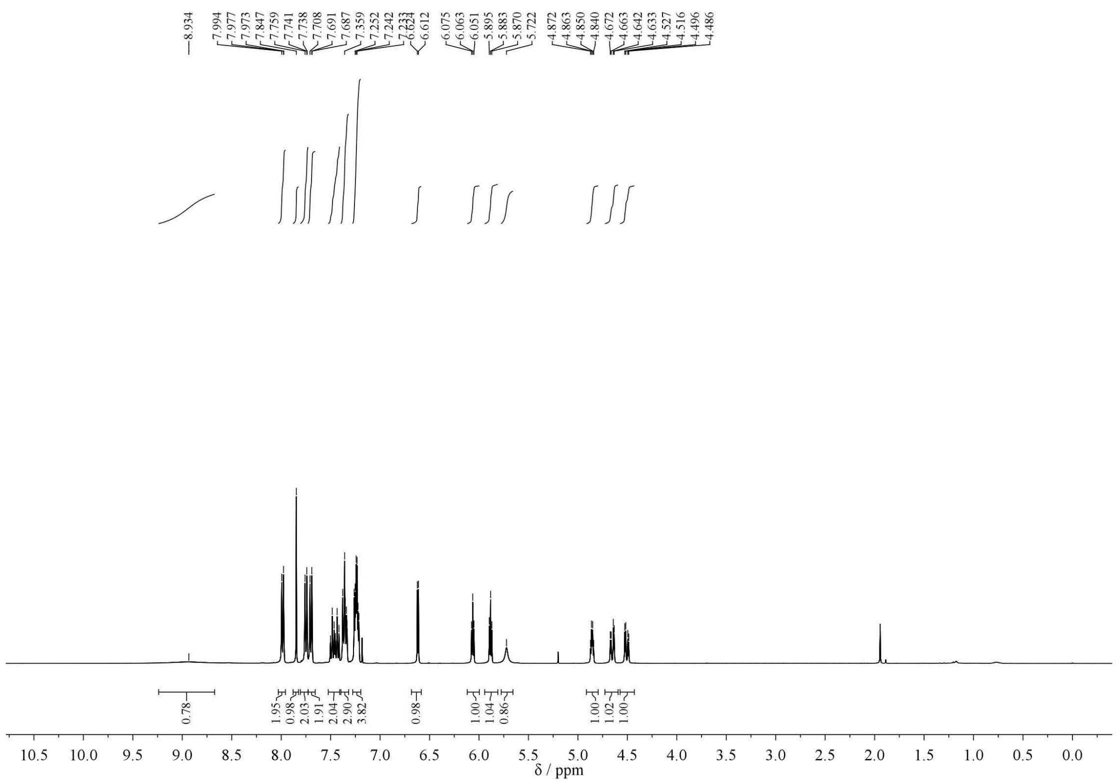

Figure S16. ^1H NMR spectrum of 2′,3′,5′-tribenzoyl-5-bromo-α-cytidine **14** in $CDCl_3$.

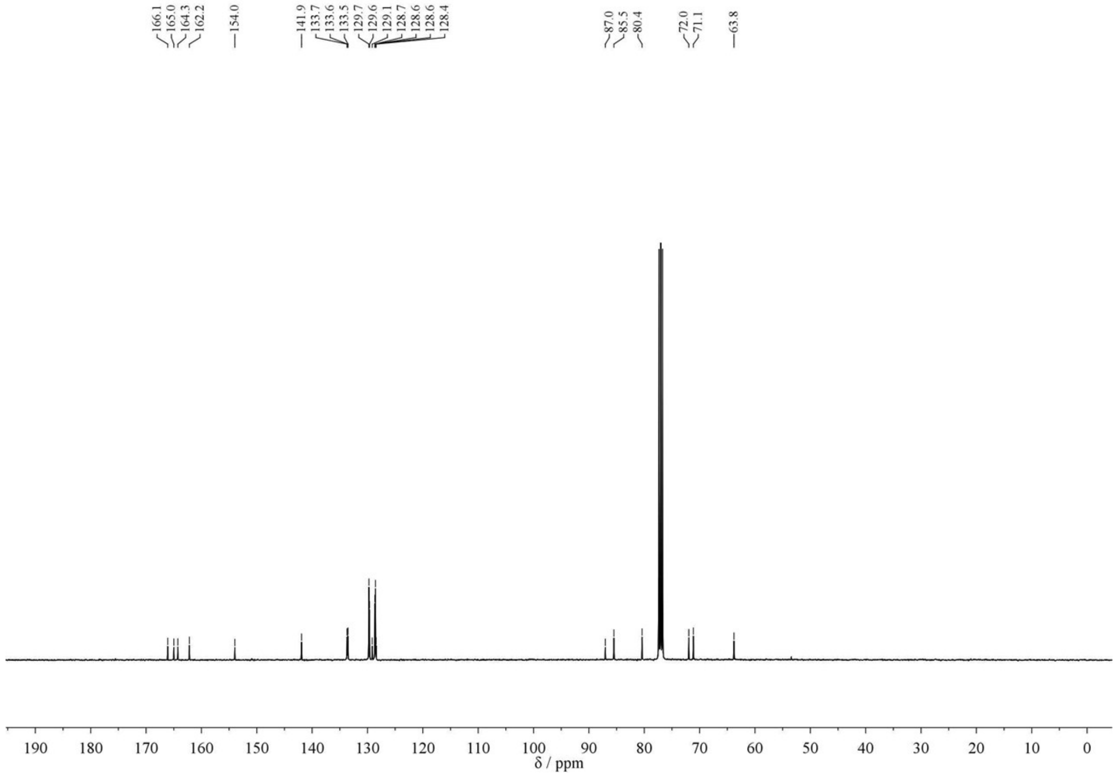

Figure S17. ^{13}C NMR spectrum of 2′,3′,5′-tribenzoyl-5-bromo-α-cytidine **14** in $CDCl_3$.

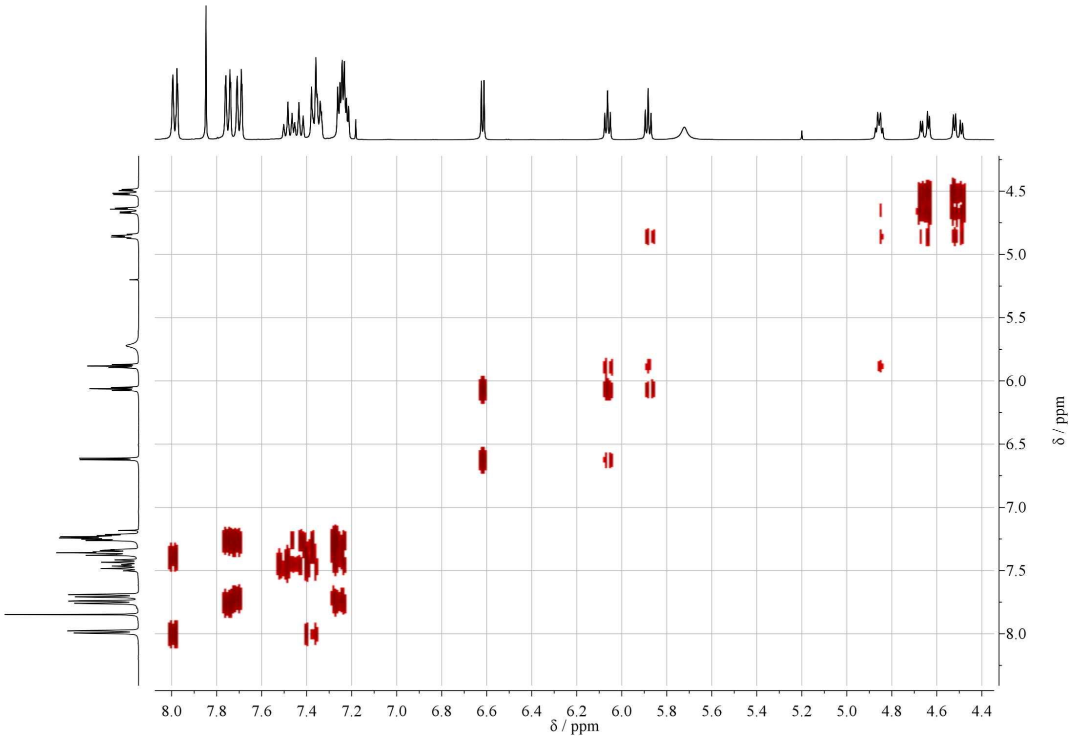

Figure S18. COSY NMR spectrum of 2′,3′,5′-tribenzoyl-5-bromo-α-cytidine **14** in $CDCl_3$.

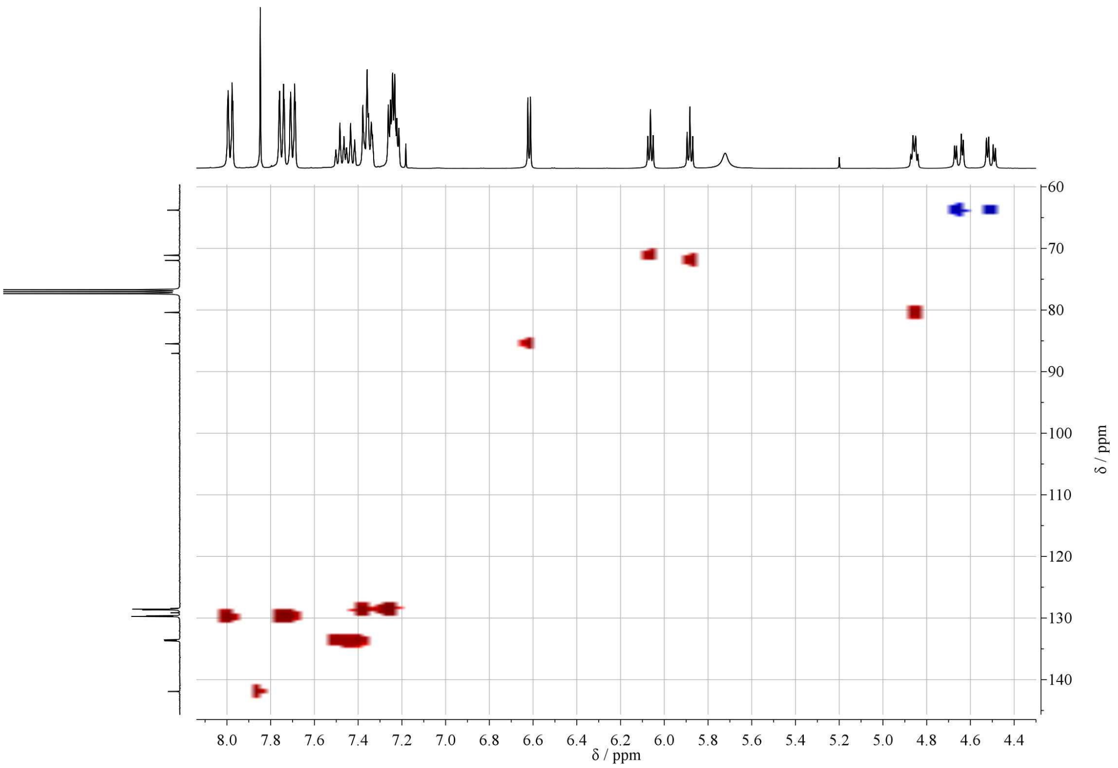

Figure S19. HSQC NMR spectrum of 2′,3′,5′-tribenzoyl-5-bromo-α-cytidine **14** in $CDCl_3$.

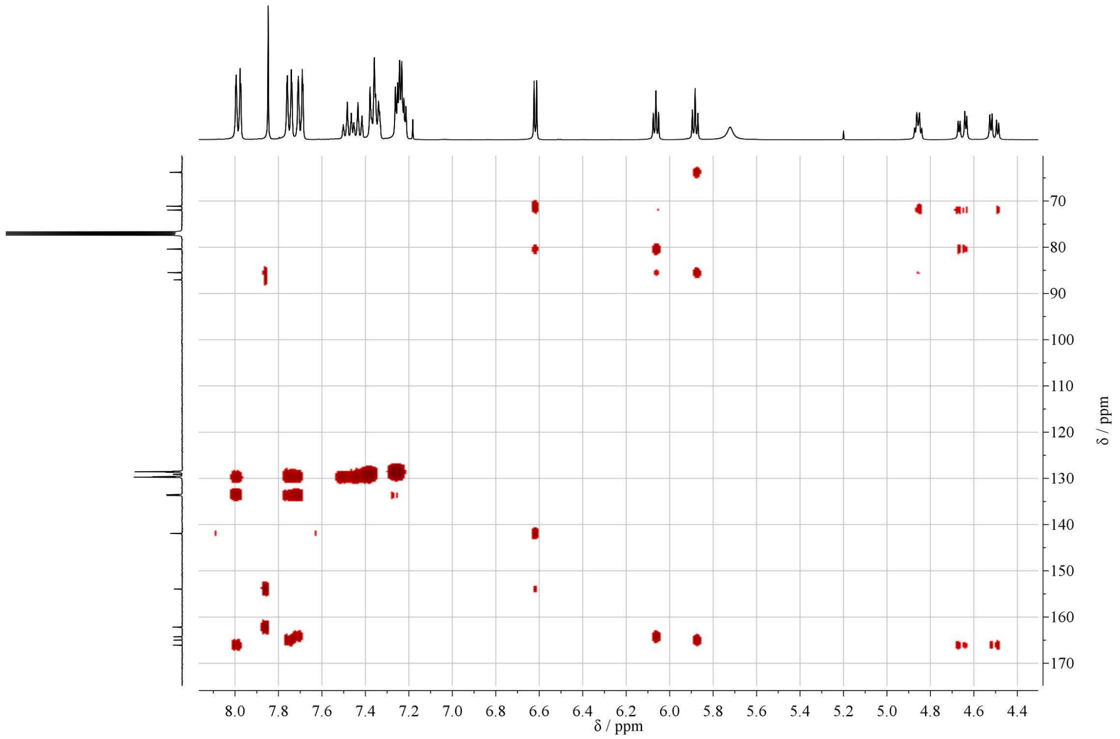

Figure S20. HMBC NMR spectrum of 2′,3′,5′-tribenzoyl-5-bromo-α-cytidine **14** in $CDCl_3$.

7. Synthesis of 2′,3′,5′-tribenzoyl-6-cyano-α-cytidine 15

2′,3′,5′-Tribenzoyl-5-bromo-α-cytidine **14** (60 mg, 0.10 mmol) was dissolved in dry DMF (5 mL) and then sodium cyanide (5.1 mg, 0.10 mmol) was added at rt. The mixture was stirred at rt and the reaction was monitored by TLC and ^{1}H NMR spectroscopy. After 31 h some unreacted starting material remained, so sodium cyanide was added (1 mg, 0.02 mmol) and left to react at rt for another 23 h (total reaction time 44 h). The colorless solution was concentrated under vacuum, before it was separated by flash column chromatography using solvent mixtures of DCM:MeOH increasing the polarity from 99:1 to 90:10 to give, after evaporation, 41 mg of the pure product **15** (yield 75%). ^{1}H NMR (400 MHz, $CDCl_3$) δ 8.53 (brs, 1H, NH), 8.03 – 7.92 (m, 2H, Bz), 7.87 (d, *J* = 8.0 Hz, 2H, Bz), 7.73 (d, *J* = 8.0 Hz, 2H, Bz), 7.41 (dt, *J* = 15.1, 7.4 Hz, 3H, Bz), 7.28 (dt, *J* = 11.3, 7.8 Hz, 4H, Bz), 7.22 – 7.12 (m, 2H, Bz), 6.73 (d, *J* = 3.7 Hz, 1H, H1′), 6.68 (brs, 1H, NH), 6.38 (s, 1H, H5), 6.11 (t, *J* = 4.0 Hz, 1H, H2′), 5.89 (dd, *J* = 8.6, 4.7 Hz, 1H, H3′), 5.10 (dt, *J* = 7.9, 3.7 Hz, 1H, H4′), 4.70 (dd, *J* = 12.1, 2.8 Hz, 1H, H5′), 4.51 (dd, *J* = 12.2, 4.3 Hz, 1H, H5″); ^{13}C NMR (101 MHz, $CDCl_3$) δ 166.2 (C=O, Bz5′), 165.1 (C=O, Bz3′), 164.7 (C=O, Bz2′), 163.9 (C4), 154.4 (C=O, C2), 133.9, 133.6, 133.3, 129.9, 129.8, 129.8, 129.4, 128.7, 128.5, 128.4, 128.4, 128.2, 125.4, 112.6 (CN), 106.6 (C5), 86.7 (C1′), 78.3 (C4′), 71.4 (C3′), 71.3 (C2′), 63.3 (C1′); ESI-LCMS (neg. m/z) 579.0 $[M-H]^-$; ESI-HRMS (pos. m/z) $[M+Na]^+$ calcd for $C_{31}H_{24}N_4NaO_8$ 603.1492; found 603.1471.

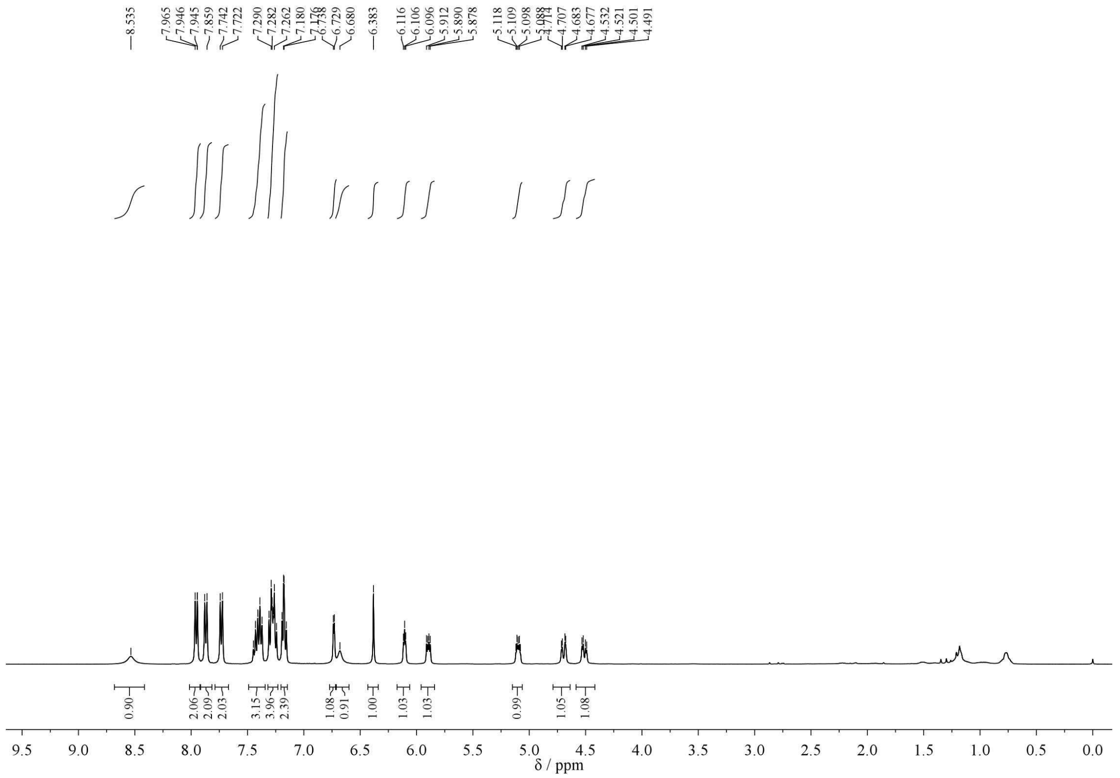

Figure S21. ^{1}H NMR spectrum of 2′,3′,5′-tribenzoyl-6-cyano-α-cytidine **15** in $CDCl_3$.

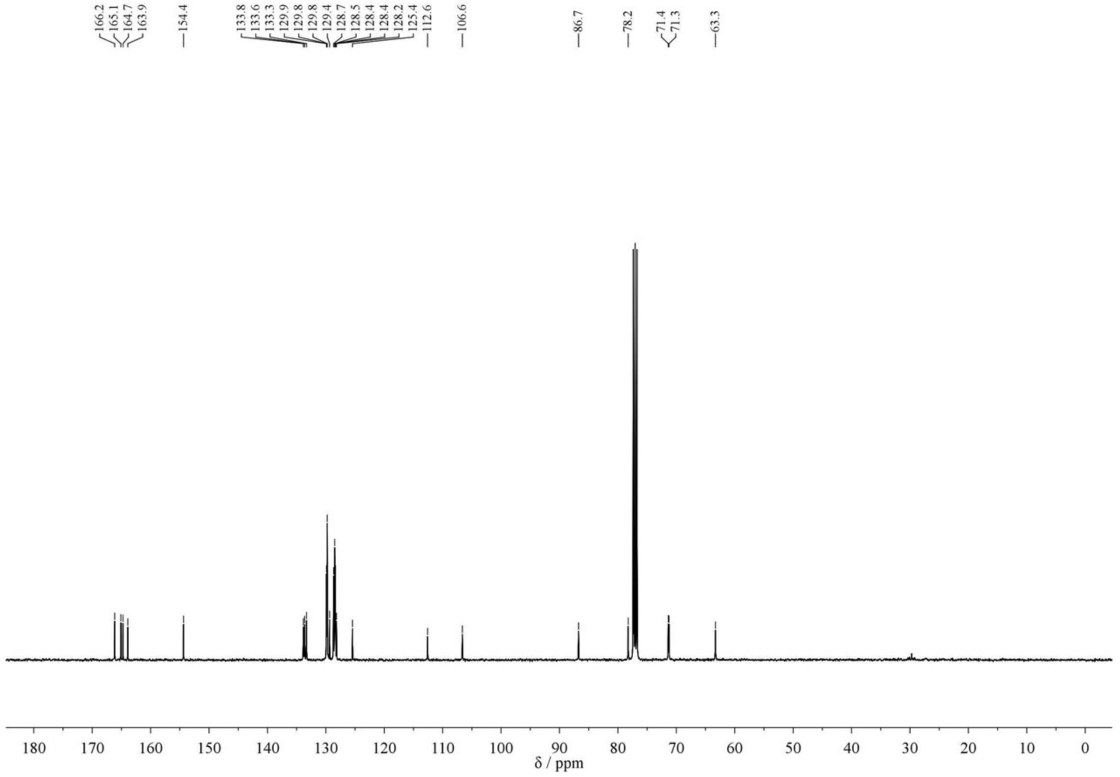

Figure S22. ^{13}C NMR spectrum of 2′,3′,5′-tribenzoyl-6-cyano-α-cytidine **15** in $CDCl_3$.

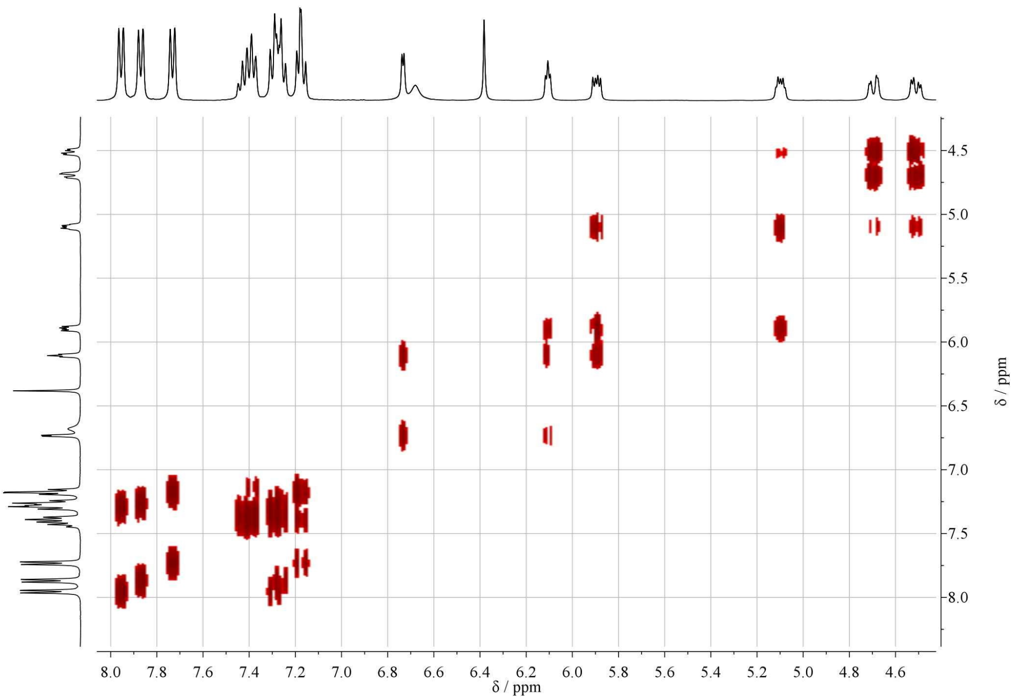

Figure S23. COSY NMR spectrum of 2′,3′,5′-tribenzoyl-6-cyano-α-cytidine **15** in $CDCl_3$.

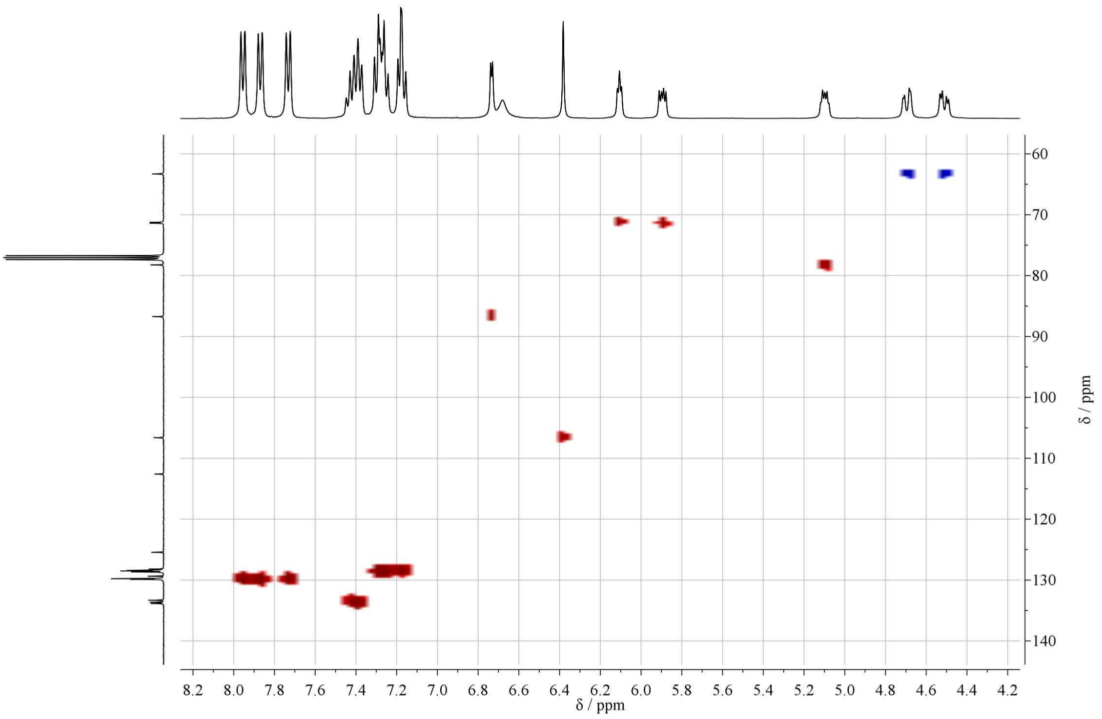

Figure S24. HSQC NMR spectrum of 2′,3′,5′-tribenzoyl-6-cyano-α-cytidine **15** in $CDCl_3$.

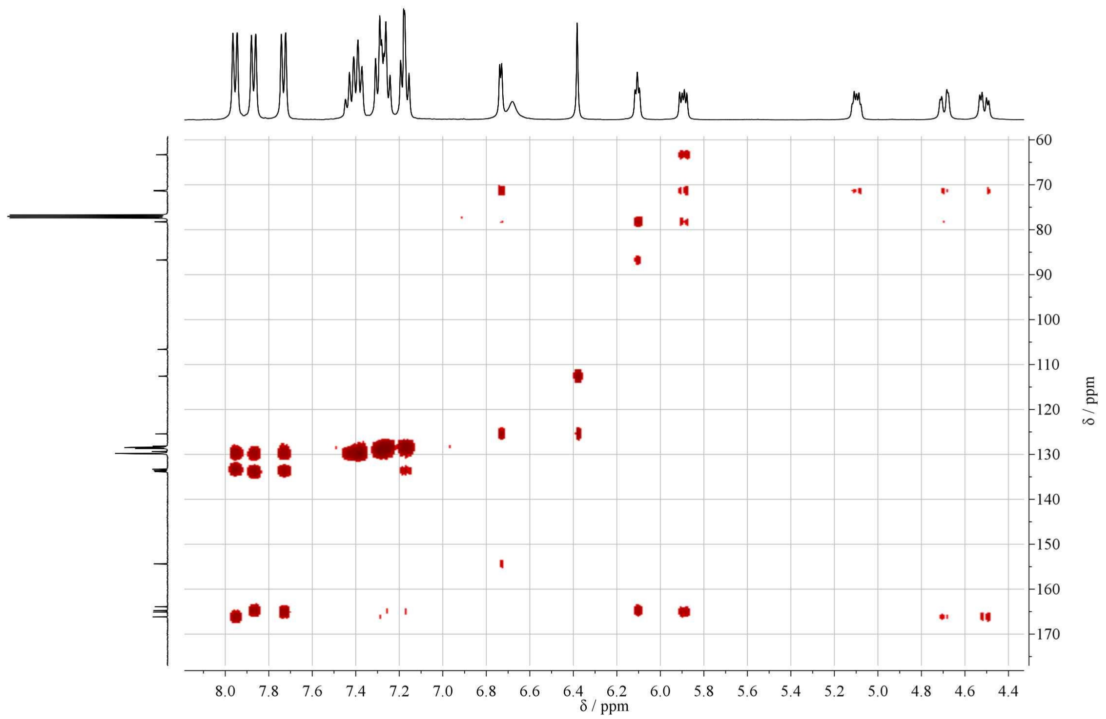

Figure S25. HMBC NMR spectrum of 2′,3′,5′-tribenzoyl-6-cyano-α-cytidine **15** in $CDCl_3$.

8. Synthesis of amide acetal 11

2′,3′,5′-Tribenzoyl-6-cyano-α-cytidine **15** (15 mg, 0.026 mmol) was dissolved in dry methanol (2 mL) and then sodium methoxide (NaOMe) (1.7 mg, 0.031 mmol) was added. This mixture was stirred at rt for 2 h and monitored by TLC, then it was evaporated and the residue was suspended in DCM and diethylether, filtered and then dried under vacuum. The white solid was dissolved in water and the pH of the solution was adjusted to 7. The solution was lyophilized to give the pure white product **11** (7 mg, yield 99%). All the analytical data were in agreement with those reported above.

References

1. A. J. Saggiomo, *J. Org. Chem.*, 1957, **22**, 1171–1175.
2. R. Faust, F. Mitzel, *J. Chem. Soc., Perkin Trans. 1*, 2000, **22**, 3746–3751
3. M. W. Powner, B. Gerland, J. D. Sutherland, *Nature*, 2009, **459**, 239-242.
